# Reporting and analysis of repeated measurements in preclinical animals experiments

**DOI:** 10.1371/journal.pone.0220879

**Published:** 2019-08-12

**Authors:** Jing Zhao, Chong Wang, Sarah C. Totton, Jonah N. Cullen, Annette M. O’Connor

**Affiliations:** 1 Department of Statistics, college of Statistics, Iowa State University, Ames, Iowa, United States of America; 2 Department of Veterinary Diagnostic and Production Animal Medicine, College of Veterinary Medicine, Iowa State University, Ames, Iowa, United States of America; 3 Independent researcher, Guelph, ON, Canada; University of Queensland, AUSTRALIA

## Abstract

A common feature of preclinical animal experiments is repeated measurement of the outcome, e.g., body weight measured in mice pups weekly for 20 weeks. Separate time point analysis or repeated measures analysis approaches can be used to analyze such data. Each approach requires assumptions about the underlying data and violations of these assumptions have implications for estimation of precision, and type I and type II error rates. Given the ethical responsibilities to maximize valid results obtained from animals used in research, our objective was to evaluate approaches to reporting repeated measures design used by investigators and to assess how assumptions about variation in the outcome over time impact type I and II error rates and precision of estimates. We assessed the reporting of repeated measures designs of 58 studies in preclinical animal experiments. We used simulation modelling to evaluate three approaches to statistical analysis of repeated measurement data. In particular, we assessed the impact of (a) repeated measure analysis assuming that the outcome had non-constant variation at all time points (heterogeneous variance) (b) repeated measure analysis assuming constant variation in the outcome (homogeneous variance), (c) separate ANOVA at individual time point in repeated measures designs. The evaluation of the three model fitting was based on comparing the p-values distributions, the type I and type II error rates and by implication, the shrinkage or inflation of standard error estimates from 1000 simulated dataset. Of 58 studies with repeated measures design, three provided a rationale for repeated measurement and 23 studies reported using a repeated-measures analysis approach. Of the 35 studies that did not use repeated-measures analysis, fourteen studies used only two time points to calculate weight change which potentially means collected data was not fully utilized. Other studies reported only select time points (*n* = 12) raising the issue of selective reporting. Simulation studies showed that an incorrect assumption about the variance structure resulted in modified error rates and precision estimates. The reporting of the validity of assumptions for repeated measurement data is very poor. The homogeneous variation assumption, which is often invalid for body weight measurements, should be confirmed prior to conducting the repeated-measures analysis using homogeneous covariance structure and adjusting the analysis using corrections or model specifications if this is not met.

## Introduction

A repeated measures design element refers to the practice of measuring the outcome on each study unit multiple times. Most frequently the multiple measurements occur over time, although other factors can be studied such as repeated exposure of individuals to changing levels of sound or light. Our interest in the approaches to the use of repeated measurements in preclinical animal experiments relate to the impact of statistical analysis on the reproducibility and reliability of results [1]. If the assumptions of statistical tests are violated, there is the potential issue that the estimates of variation are incorrect and type I and II errors are not appropriately controlled. There has been concern that increased type I error rates and reduced variation estimates can contribute to the reproducibility crisis in biomedical research due to the preference to publish significant results [2, 3]. However, equally important is the fact that variance and type II error rate inflation can mean studies with important findings might remain unpublished due to publication bias. The solution to these issues is appropriate analysis based on the underlying data structure that minimizes both types of errors and provides accurate estimates of precision.

Repeated measures is a very common study design element in preclinical animal research. In a survey of 200 preclinical animal experiments, 58 manuscripts contained language that described the repeated measurement of at least one outcome on study subjects [4]. Inclusion of a repeated measurements design element can be desirable for a variety of reasons. Repeated measurement of outcomes is often included if the impact of the factor, time, or changes in the effect of treatment over time are of explicit interest to the investigator. Studying multiple outcomes for each subject might also allow investigators to reduce subject-to-subject and within-subject variation in the investigation of the relative effects of different treatments. Reduced variability might increase the study’s power, and this is another rationale for implementing repeated measurements [5].

Repeated measures analyses are often conducted with either repeated measures ANOVAs or multilevel modelling. Repeated measures ANOVAs are common methods that rely on balanced data and can be implemented with PROC GLM in SAS and ezANOVA in R. In contrast, multilevel models, which are usually implemented with Proc MIXED in SAS, lme4 or nlme using R, or statsmodels for Python, allow for unbalanced data but require more elaborate fitting algorithms and model specifications than repeated measures ANOVAs. In both models, the specification of the variance covariance structure, which models the covariation among measurements from the same subject, is the key step in the analysis of repeated measures. There are several variance covariance structures available for selection by analysts, and many statistical software procedures implement the compound symmetry structure by default. The compound symmetry variance covariance structure assumes that all the variances are equal and all the covariances are equal. These assumptions, however, might be incorrect and a different structure might better describe the variance between subjects and covariation within subjects. It is important to study the impact of making these incorrect assumptions and choosing the wrong variance covariance structure in a repeated measures analysis.

Given the high frequency of repeated measurements design in preclinical animal experiments and analysis with different approaches, our objectives in this study were to (i) summarize how current researchers report studies with repeated measurements design; (ii) assess the impact of choice of analysis approach of repeated measures using simulation modelling on the *p*-value distributions, the type I, and type II error rates. For each of these aims, the knowledge gained could be used to inform educational activities for researchers that promote approaches to reporting and analysis that maximize the value obtained from animals used in preclinical experiments.

## Materials and method

### Eligible studies and study selection

This project used a subset from 200 manuscripts used in another study that assessed the presence of seven design elements in preclinical animal experiments. The 200 studies are preclinical experiments on brain trauma and toxicology, many with repeated measure design elements that were identified for a different study. Eligible studies included described primary research of a single comparative pre-clinical animal experiment [4]. Of the 200 studies, 100 studies were randomly selected from eligible studies and characterized for the design features used by the authors. This process included assessment of the design by two independent reviewers. The studies used here are those that used a repeated measures design element. Fifty-eight of the 200 studies were classified as containing language that suggested repeated measurement of the outcome i.e., a repeated measures design element. Two approaches to recognition of a repeated measures design element were used. A repeated measures design element was one where the investigators described a process of repeated measurements of outcomes on a study unit such as measurement of animal body weight weekly for 10 weeks. Alternatively, if the investigators described a statistical method that would be considered a repeated measures statistical analysis, such as “a repeated measures ANOVA was conducted”, this was also used as evidence for a repeated measures design. These two criteria were used to select all studies with repeated measures design elements (*n* = 58: designated for “repeated measures (RM) studies”). Within the RM studies, a smaller subset of studies (*n* = 31) with body weight as the outcome (specified with the prefix “BW-”) was also formed. Additionally, we differentiated repeated measurement from pseudoreplication, another form of replication e.g., repeated measurement of the number of cells on the same histopathology slide [6]. The distinction between the two forms of replication is that, unlike pseudoreplicates, repeated measurements are associated with another study factor, such as time, that can be included in the data analysis.

### Aim 1: Assessment of the rationale, approaches to analysis, and approach to the reported results of analysis

We assessed the investigators’ rationale for inclusion of the repeated measures design element by determining if the investigators provided either a description of treatment over time in the objectives or a statement of the higher power with a repeated-measure analysis in comparison with single time point study (e.g., ANOVA at one time point) in the introduction section.

For the assessment of approaches to analysis, for all studies, we extracted the number of treatments and the number of time points assessed. We assessed if the 58 studies used repeated-measures analysis (RMA) or not (NoRMA) and if the investigators reported assessing the normality of residual errors and homogeneity of variance. Key phrases indicating the repeated measures statistical analysis included “repeated-measures ANOVA”, “repeated measures analysis of variance”, and “MIXED model with subject as a random effect”, etc. Mentioning of assessment of normality or homogeneity of variance for at least one outcome was designated as “yes”.

With respect to reporting the results, we determined if the investigators reported the following when applicable: overall repeated measures model effect by specifying either main effect including treatment effect, time effect, or treatment-by-time interaction, and test for treatment at single time points. We also evaluated if the investigator presented the observed data from each group at each time point in either a figure or table and whether the precision was reported. As many investigations have multiple outcomes, a positive response (yes) was recorded if the investigators provided the information of interest for at least one outcome. We also examined the association between the meta-factors (year of publication, the first 24 months’ citations, journal impact factor in the published year, and journal impact factor in 2016) and the use of RMA analysis in the subset of 31 studies that reported the effect of treatments on body weight. A detailed description of these variables is available in Supplementary materials.

### Aim 2: The impact of analysis approach on tests for treatment effects at single time points

Aim 2 sought to evaluate the impact of different analysis approaches on repeated measures data. This aim first required setting the parameters (the mean, the SD and the covariance among subjects) for the observations in the simulation datasets. These parameters were then combined with other simulation settings to create multiple datasets which varied in the sample size, treatment effect on the outcome and the covariation among measurements from the same subject. The impact of different approaches to statistical analysis were then evaluated. More detail about these steps is provided below.

#### Parameter identification for simulation

With the purpose of empirical simulation, figure 2 of Cho et al. [6], which presented data for the effect of three dietary treatments on the body weight of 11-12 mice per group weekly for 29 weeks, was selected as the basis for the simulation. The means and mean standard errors (SEMs) at five time points (week 1, 7, 14, 21 and 28) for each treatment combination were extracted using Plot Digitizer software version 2 [7]. The standard deviations (SDs) were obtained by multiplying the SEM by the square root of the sample size of each group, which was set to 12.

Two simulations were conducted corresponding to a null model and an alternative model, for assessment of type I error rates, type II error rates and SEM estimation. Under the null model which equates to no treatment effect, the population mean parameter at each time point was set as the average of the means of the three treatments at week 1, 7, 14, 21 and 28 from Cho et al. [6]. The SDs used for each time point and treatment combination were those extracted from the Cho et al. [6] as described above. The population parameters (means and SEMs) of the alternative model at each time point were directly extracted from Cho et al. [6]. The only difference in the simulation parameters between the null and the alternative model was the mean parameters, that is the null model has no group differences in contrast to the alternative simulation model. All parameters used for simulation setting are summarized in Table 1.

**Table 1:** Parameters used for simulations approximated from Cho et al. (2013).

Treatment	Time	Mean in the null simulation	Mean in the alternative simulation	SEM	SD
HFol-Hfol	week 1	160	166.45	7.97	27.57
HFol-RV	week 1	160	165.27	6.79	23.48
RV-RV	week 1	160	149.92	6.00	20.76
HFol-Hfol	week 7	480	478.77	8.26	28.59
HFol-RV	week 7	480	492.34	12.98	44.92
RV-RV	week 7	480	468.14	11.21	38.80
HFol-Hfol	week 14	630	615.83	17.70	61.26
HFol-RV	week 14	630	654.19	21.54	74.53
RV-RV	week 14	630	619.96	15.34	53.09
HFol-Hfol	week 21	724	697.41	19.48	67.38
HFol-RV	week 21	724	752.89	26.85	92.91
RV-RV	week 21	724	721.02	16.23	56.15
HFol-Hfol	week 28	800	764.83	23.90	82.70
HFol-RV	week 28	800	835.06	33.05	114.35
RV-RV	week 28	800	790.21	15.64	54.11

HFol-HFol: 10-fold folate (HFol) diet for both mother and pup; HFol-RV: 10-fold folate (HFol) diet for mother, recommended vitamin is for pup; RV-RV: recommended vitamin diet for both mother and pup. Data were approximated using Plot Digitizer. Mean in the alternative simulation: directly extracted mean value of each treatment and time combination from Cho et al. [6]. Mean in the null simulation: average mean value of three treatments at week 1, 7, 14, 21, and 28 from mean value in the alternative simulation. SEM was directly extracted from Cho et al. [6] and SD denotes for standard deviation calculated by $SE*\sqrt{12}$ (sample size per group).

#### The simulation procedure

Two sets of simulations were conducted using the parameters in Table 1. We varied the following conditions in the null and alternative model to create the underlying data:

1. Two heterogeneous population variance covariance structures; heterogeneous compound symmetry (heterogeneous_CS) as in Eq (1) or heterogeneous first order autoregressive (heterogeneous_AR) as in Eq (2). The rationale for creating a dataset with heterogeneous variance is that the data from Cho et al appear to show that the variance of the outcome, body weight, increases over time, which implies heterogeneous variance (Table 1 and S1 Fig). The covariance of heterogeneous compound symmetry assumes that the same correlation *ρ* between observations at any two pairs of time points for the same subject. A first-order autoregressive covariance assumes that correlations *ρ* decline exponentially with distance, which means that as measurements get farther apart, they are less correlated. Both structures of covariance are thought to frequently occur in repeated measures data.
2. three sample sizes for each group (*n* = 3, 6, 12 per group);
3. four correlation coefficient *ρ* values (*ρ* = 0.1, 0.3, 0.6, 0.9), where correlation coefficients were specified with regard to time points

Multivariate normal random variables with the specific mean and variance covariance structures were simulated for each combination (2 × 3 × 4) of conditions 1,000 times.

#### Analysis on simulated data

Using the 2 × 3 × 4 simulated data output for either null or alternative model, three statistical analysis approaches were conducted:

i Repeated measures analysis (RMA) where the variance covariance structure in the model correctly represents the underlying data.
  For the data simulated with heterogeneous compound symmetry, the analysis would be implemented using heterogeneous compound symmetry variance covariance structure (heterogeneous_CS) as in Eq (1); For the data simulated by heterogeneous first order autoregressive, the correctly specified structure in the analysis would be the heterogeneous first order autoregressive variance covariance structure (heterogeneous_AR) as in Eq (2);
  $$\begin{array}{c} heterogeneous\;compound\;symmetry=[\begin{array}{ccccc} \sigma_{1}^{2} & \sigma_{1}\sigma_{2}\rho & \sigma_{1}\sigma_{3}\rho & \sigma_{1}\sigma_{4}\rho & \sigma_{1}\sigma_{5}\rho \\ & \sigma_{2}^{2} & \sigma_{2}\sigma_{3}\rho & \sigma_{2}\sigma_{4}\rho & \sigma_{2}\sigma_{5}\rho \\ & & \sigma_{3}^{2} & \sigma_{3}\sigma_{4}\rho & \sigma_{3}\sigma_{5}\rho \\ & & & \sigma_{4}^{2} & \sigma_{4}\sigma_{5}\rho \\ & & & & \sigma_{5}^{2} \end{array}] \end{array}$$ (1)
  $$\begin{array}{c} heterogeneous\;first\;order\;autoregressive=[\begin{array}{ccccc} \sigma_{1}^{2} & \sigma_{1}\sigma_{2}\rho & \sigma_{1}\sigma_{3}\rho^{2} & \sigma_{1}\sigma_{4}\rho^{3} & \sigma_{1}\sigma_{5}\rho^{4}\\ & \sigma_{2}^{2} & \sigma_{2}\sigma_{3}\rho & \sigma_{2}\sigma_{4}\rho^{2} & \sigma_{2}\sigma_{5}\rho^{3}\\ & & \sigma_{3}^{2} & \sigma_{3}\sigma_{4}\rho & \sigma_{3}\sigma_{5}\rho^{2}\\ & & & \sigma_{4}^{2} & \sigma_{4}\sigma_{5}\rho \\ & & & & \sigma_{5}^{2} \end{array}] \end{array}$$ (2)
  The variance covariance matrices are symmetric with the value in diagonal representing the variance at each time point and values off diagonal denoting covariance of pairwise time points. Where $\sigma_{1}^{2},\sigma_{2}^{2},\sigma_{3}^{2},\sigma_{4}^{2},and\;\sigma_{5}^{2}$ on the diagonal represent variance at five time point of the two structure above, and different subscript of *σ*^2^ designates unequal variance $\left(\sigma_{1}^{2}\neq \sigma_{2}^{2}\neq \sigma_{3}^{2}\neq \sigma_{4}^{2}\neq \sigma_{5}^{2}\right)$. For off diagonal value: in the heterogeneous_CS structure as in Eq (1), *ρ* (0 ≤ *ρ* ≤ 1) is the constant correlation of pairwise time points, and *σ*_*i*_*σ*_*j*_*ρ*(*i* ≠ *j*) is the covariance between *time*_*i*_ and *time*_*j*_ (e.g., *σ*_1_*σ*_2_*ρ* is the covariance between *time*_1_ and *time*_2_ of the same subject); in the heterogeneous_AR structure as in Eq (2), *ρ*^(*j*−*i*)^ are the correlations between *time*_*j*_ and *time*_*i*_, and *σ*_*i*_*σ*_*j*_*ρ*^(*j*−*i*)^ represents covariance between *time*_*i*_ and *time*_*j*_ (e.g., *σ*_1_*σ*_3_*ρ*^(3−1)^ is the covariance between *time*_1_ and *time*_3_ of the same subject).
ii Repeated measures analysis (RMA) where the variance covariance structure in the model does not correctly represent the underlying data due to equal variance assumption.
  For the data simulated by heterogeneous compound symmetry variance covariance structure as in Eq (1), the incorrectly specified variance covariance structure was homogeneous compound symmetry (homogeneous_CS) as in Eq (3). This analysis approach is commonly referred to as the compound symmetry (CS) or exchangeable structure and is the default for most software procedures or packages. For data simulated by heterogeneous first order autoregressive variance covariance structure as in Eq (2), the incorrectly specified variance covariance structure in the analysis was homogeneous first order autoregressive (homogeneous_AR) as in Eq (4).
  $$\begin{array}{c} homogeneous\;compound\;symmetry=\sigma^{2}[\begin{array}{ccccc} 1 & \rho & \rho & \rho & \rho \\ & 1 & \rho & \rho & \rho \\ & & 1 & \rho & \rho \\ & & & 1 & \rho \\ & & & & 1 \end{array}] \end{array}$$ (3)
  $$\begin{array}{c} homogeneous\;first\;order\;autoregressive=\sigma^{2}[\begin{array}{ccccc} 1 & \rho & \rho^{2} & \rho^{3} & \rho^{4}\\ & 1 & \rho & \rho^{2} & \rho^{3}\\ & & 1 & \rho & \rho^{2}\\ & & & 1 & \rho \\ & & & & 1 \end{array}] \end{array}$$ (4)
  Where *σ*^2^ in the diagonal represents the constant variance at each five time point of both structures. For off diagonal value: in the homogeneous_CS structure as in Eq (3), *ρ* (0 ≤ *ρ* ≤ 1) is the constant correlation between pairwise time points, and *σ*^2^ *ρ* represents the constant covariance; in the homogeneous_AR as in Eq (4), *ρ*^(*j*−*i*)^ are the correlations between *time*_*j*_ and *time*_*i*_, and *σ*^2^*ρ*^(*j*−*i*)^ denotes covariance between *time*_*i*_ and *time*_*j*_ (e.g., *σ*^2^*ρ*^(4−1)^ is the covariance between *time*_1_ and *time*_4_ of the same subject).
iii separate ANOVA at each time point.
  There is no variance covariance structure specified across all time points in this method. There is no overall analysis for data across all time points, instead, individual ANOVA at each time point is implemented using data only at that time point respectively.

For analyses (i) and (ii), the PROC MIXED procedure in SAS software (version 9.4; SAS Institute Inc., Cary, NC) was used. Contrast statements were used to obtain the treatment effect at each time point from the RMA. The PROC GLM procedure was used to perform separate ANOVA analysis at individual time points in (iii). The distributions of *p*-values at each time point for the three analyses approaches were then plotted and compared based on results from 1000 simulations. The impact of the changing conditions on the type I and type II error rate was calculated by comparing the proportion of *p*-values falling below 0.05. Under the null model, an analysis procedure that controls type I error correctly is expected to generate a *p*-value distribution that is uniformly distributed from 0 to 1. It is expected that 5% of observations will fall between 0.05 and 0, if alpha (type I error rate) is set as 0.05. All simulation and analysis code are publicly available in Github https://github.com/jingzhao19/Plos-one-i2019.git.

## Results

### Aim 1: Assessment of the rationale, approaches to analysis, reporting results and association with meta-factors

Fifth-eight of 200 articles met the criteria of repeated measures design element [4], i.e., used language that suggested repeated measurement of the outcome on study subjects. As summarized in Table 2, only three of the 58 studies included a rationale of a repeated measurement of the outcome. All three studies reported explicit interest in the treatment over time in the objectives. Of the 58 studies, 23 studies reported using a repeated-measures analysis (RMA) and 35 studies did not (NoRMA). Twenty of the 23 studies used the term “repeated-measures” to define RMA studies. Analysis of Variance (ANOVA) at a single time point was the most common statistical approach employed in the NoRMA studies (31/35).

**Table 2:** The reporting characteristics of 58 biomedical animal experiments with repeated measurement of an outcome.

Category	Examined factors	RMA studies (*n* = 23)	NoRMA studies(*n* = 35)
	Body weight as one of outcomes in repeated-measures	9	22
Rationale	Provided a rationale for including a repeated measures design	3	0
Assumptions	Conducted a test for normality	3	6
	Conducted a test for equality of variance	2	10
Repeated measures model results**	Treatment effect	20	NA
	Time effect	15	NA
	Treatment-by-time interaction effect	17	NA
Single time point result**	Tested at each time point using raw data	13	24
	Tested at all time points	13	14
	Tested at specific time points only	0	12
	No tests conducted at single time point	11	18
	Tested using the derived data body weight gain (= weight at end time point weight at initial time point)	0	14
Data presentation**	Figure only	15	12
	Table only	2	14
	Figure & table	4	6
	Precision estimate included	21	31

RMA: repeated measure analysis. NoRMA: nonrepeated measures analysis. NA: Not applicable.

**As multiple outcomes can occur within a single study, these categories are not mutually exclusive.

For both RMA and noRMA, as shown in Table 2, a minority of studies reported assessing the normality or homogeneity variance, three (3/23: 13%) and two (2/23: 9%) respectively in RMA, six (6/35: 17%) and ten (10/35: 29%) respectively in noRMA. Precision of estimation was frequently reported in both RMA (21/23) and noRMA (31/35). RMA studies were more likely to present data using figures (15/23), while noRMA randomly present data as figures (12/35) or as tables (14/35). A clear difference was observed in the reporting of the overall repeated measures model between the RMA and NoRMA studies. RMA studies usually reported an overall model effect (main effects of treatment, time, or interaction between treatment and time) and could subsequently also report the effect of treatment at single time points. However, studies that did not do RMA, only reported analysis conducted at individual time points. This difference is reflected in the data summarized in Table 2. RMA studies presented overall results mainly by treatment effect (20/23) or treatment-by-time interaction (17/23) and followed with testing for treatment at individual time points (13/23). All 13 studies that reported the treatment effect of RMA model also reported the assessment of the treatment effect at each time point. In the noRMA category, 69% (24/35) articles directly reported testing for a treatments effect either at specific time points or all measured time points, for at least one outcome. Additionally, fourteen of 35 studies converted the data to a single overall metric such as body weight gain (body weight at the endpoint- initial body weight) e.g., [8–11].

Meta factor analyses were applied only in the Bw-dataset in which body weight was the common outcome S3 Table. The Bw-dataset was also examined either using a repeated measure analysis (RMA-BW) or not (NoRMA-BW), and differences between RMA-BW and NoRMA-BW were detected in the year of publication, journal impact factor in the published year, and the first 24 months’ citations S4 Table and S2 Fig. In summary, articles that used RMA tended to be published more recently, in journals with higher impact factor in the published year, and to have more citations in the first 24 months’ post publication.

### Aim 2: The impact of analysis approach on type I and type II error of treatment effects estimated at single time points

Here, we only present two representative scenarios for null and alternative simulation analysis. The results of the remaining 46 scenarios as extended information are available in GitHub https://github.com/jingzhao19/Plos-one-i2019.git. Each simulation setting was analyzed by three approaches (i,ii,iii) as described in materials and methods. The distributions of *p*-values for testing

1. the overall treatment effect (only applicable for analysis approaches i and ii),
2. treatment-by-time interaction (only applicable for analysis approaches i and ii), and
3. treatment effect at each time point

were identified.

**Fig 1:**
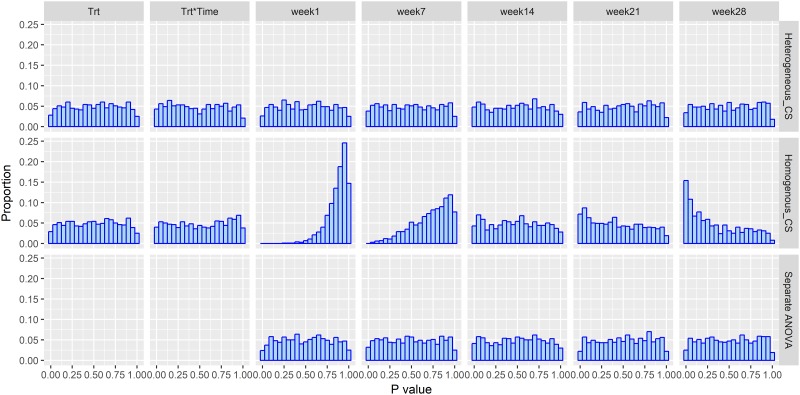
Data analysis using heterogeneous_CS as in Eq (1), homogeneous_CS in Eq (3), and separate ANOVA based on the null model simulation with *ρ* = 0.3, *n* = 12 and a heterogeneous_CS variance covariance structure. Trt: overall treatment effect. Trt*Time: Treatment by Time interaction. Week 1, Week 7, Week 14, Week 21, and Week 28: *p*-value distribution at each time point. Proportion = count per bin/1000.

**Fig 2:**
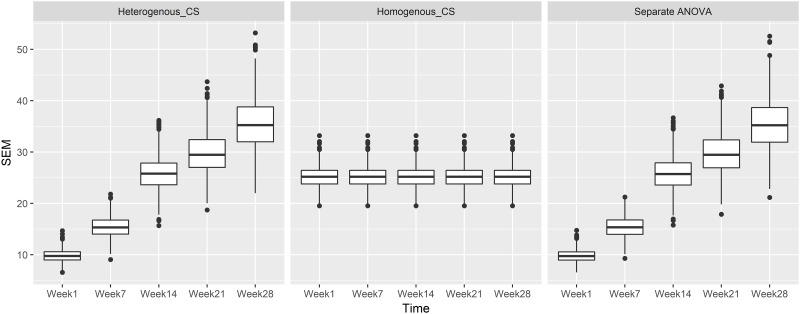
SEM estimation using heterogeneous_CS as in Eq (1), homogeneous_CS in Eq (3), and separate ANOVA based on the null model simulation with *ρ* = 0.3, *n* = 12 and a heterogeneous_CS variance covariance structure. Week 1, Week 7, Week 14, Week 21, and Week 28: SEM distribution for pairwise comparisons between treatments at each time point. Proportion = count per bin/1000.

The impact of incorrectly specifying the variance structure when there is no association between treatment and the outcome with simulation under compound symmetry covariance structure, *ρ* = 0.3 and *n* = 12 is shown in Fig 1. The expected uniform distribution of the *p*-value is observable in the first row of Fig 1. The row one data presented in Fig 1 represents an analysis that correctly models the variance structure of the repeated measurement data i.e., heterogeneous compound symmetry variance covariance structure as in Eq (1). Similarly, the uniform distribution of the *p*-value is observable when the repeated measurements analysis is ignored and separate ANOVAs for the treatment effects are conducted at each time point as shown in row three of Fig 1. By comparison, when the true heterogeneous variance structure is ignored, and instead the modeling approach assumes that the variation in the body weight is constant over time i.e., the homogeneous variance assumption, the *p*-value distribution is far from the expected uniform distribution, especially at the most distal time points, week 1 and week 28. In particular, the *p*-value distribution at “week 28” is stochastically smaller (peaked at smaller *p*-values and skewed to the right) than the expected uniform distribution. The proportion of *p*-values < 0.05 is 22.5% with homogeneous_CS as in Eq (3) based on the simulation results which indicates a much larger than expected type I error rate. This result is associated with the inappropriate calculation of SEM (the standard error for comparing treatment means) and estimated SEM based on the homogeneous_CS model. As present in Fig 2, the SEM across time in heterogeneous_CS and separate ANOVAs exhibited the same SEM trend, hence similar test results are identified. Alternatively, the estimation of SEMs from homogeneous_CS as in Eq (3) was constant across five time points, which is not consistent with the fact that the variances change over time. Due to the averaging effect across all time points, the strongest influence of the inappropriate SEM happened at distal time points such as week 1 with left skewed, week 28 which was right skewed.

**Fig 3:**
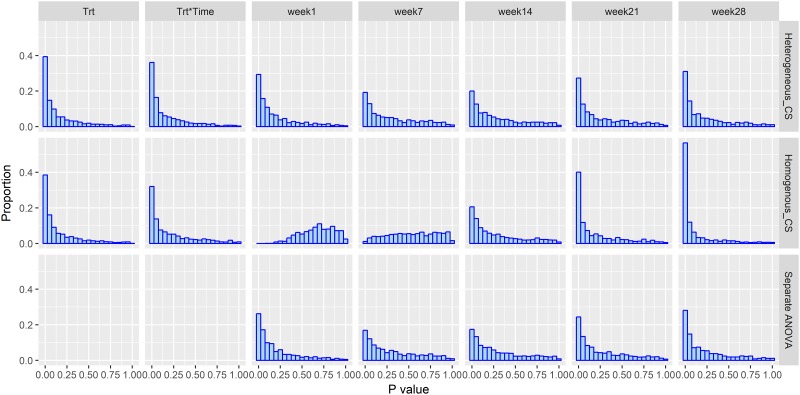
Data analysis using heterogeneous_CS as in Eq (1), homogeneous_CS in Eq (3), and separate ANOVA based on the alternative model simulation with *ρ* = 0.3, *n* = 12 and a heterogeneous_CS variance covariance structure. Trt: overall treatment effect; Trt*Time: the overall treatment-by-time interaction; week 1, Week 7, Week 14, Week 21, and Week 28: *p*-value distribution at each time point. Proportion = count per bin/1000.

**Fig 4:**
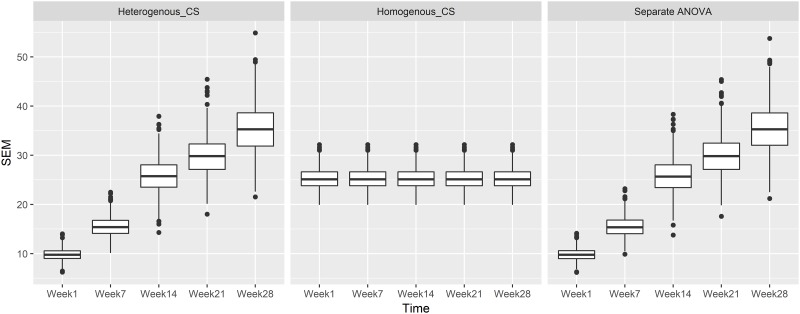
SEM estimation using repeated measures analysis with either heterogeneous_CS as in Eq (1), homogeneous_CS in Eq (3), and separate ANOVA analysis approaches at each time point based on the alternative model simulation with *ρ* = 0.3, *n* = 12 and a heterogeneous_CS variance covariance structure. Week 1, Week 7, Week 14, Week 21, and Week 28: SEM distribution for pairwise comparison between treatments at each time point. Proportion = count per bin/1000.

The results for the alternative model, i.e., a treatment effect does exist, the impact of misspecification of the variance structure for the compound symmetry covariance structure, *n* = 12 per group and *ρ* = 0.3 are presented in Fig 3. Focusing first on the correctly specified analysis in the first row of Fig 3, we can see that the *p*-values distribution is skewed to the left as expected. In the 2nd row of Fig 3, the impact of misspecification of the variance structure varies by time point. In the early weeks 1 and 7, the flat and right skewed *p*-value distributions indicate lower power (increased type II error). For the important endpoints, the incorrectly specified homogeneous variance structure is more powerful than the other two analyses methods at later time points (e.g., week 28). For example, at week 28, the proportion of *p*-values < 0.05 is 39.8% with heterogeneous variance based analysis, compared to 63.7% with the homogeneous based analysis. This should not be construed as a rationale for making the incorrect assumption as the simulation of the null model indicated homogeneous does not control type I error correctly making the approach invalid. As shown in Fig 4, the SEM across time increased in heterogeneous_CS as in Eq (1). The separate ANOVA model had similar SEM estimation with the heterogeneous_CS structure, which was used to simulate the dataset. However, the SEM estimation based on homogeneous_CS as in Eq (3) has a completely different trend (constant SEM), thus the endpoint become extraordinarily significant and less significant at week 1 and week 7.

Similar results, differing only in magnitude were observed for all scenarios evaluated and these are reported in the GitHub https://github.com/jingzhao19/Plos-one-i2019.git for the scenarios with different correlations, sample sizes, and covariance structure. The comparison of the separate ANOVA and the heterogeneous variance repeated measures approach, suggests that separate ANOVA is less powerful than testing the time effect within the repeated measurements analysis especially at the endpoint week 28. As the correlation between time points *ρ* increases, the *p*-value distribution becomes stochastically larger (less powerful). As sample size increased, the *p*-value distribution becomes stochastically smaller (more powerful).

## Discussion

Repeated measures on the outcome is a commonly used design element in preclinical animal experiments [4]. The rationale for incorporating a repeated measurement design element into an experiment is generally considered to be of interest in a treatment-by-time interaction or effective control of subject-to-subject variation [5]. We found that a surprisingly large number of studies that collected repeated measurement data did not incorporate repeated measurements statistical analysis (35 of 58). It would be interesting to understand the rationale for collecting measurements of the outcome over time; however few authors provided a clear rationale for the design element. Some investigators were clearly interested in the effect at multiple time points while others were only interested in the endpoint as evidenced by use of the weight change outcome. Both the PREPARE guidelines and the ARRIVE guidelines recommended a complete statement of objectives be included in preclinical animal experiments [12, 13], and this would imply inclusion of the rationale for a repeated measures design.

An interesting finding was the number of noRMA studies that used weight change as the outcome yet reported measuring the outcome at multiple time points, examples include [9, 11, 14]. It is unclear if collection of the unused measurements required additional handling or procedures that could have adversely impacted the welfare of the experimental animals. Some repeated outcomes, like food intake do not require additional handling, and therefore additional data collection is not associated with animal welfare, while others such as body weight might. The goal of minimization of stress and unnecessary handling of experimental animals would imply that if the pre-planned analyses did not require multiple measurements then investigators should avoid measurement for measurement’s sake, if measurement places stress on the animals [15–17].

We would propose that the rationale for repeated measurements design should be included in the statement of objectives as it provides a basis for the sample size and additional justification animal handling procedures. If repeated measurement of the outcome is not needed to answer the study hypothesis, then it might be best to not collect the data for animal welfare reasons. When the data are collected, a repeated measurements analysis should be utilized as it provides the greatest power to detect treatment effects and provides comprehensive information from the study design.

After the rationale is clarified, a comprehensive description of the study design serves as a road map for interpreting the study. This study design description should include a description of the type of design used, a description of how the study was conducted, and each factor involved in the experiment because the justification for the analysis lies not in the data collected, but in the manner in which the data were collected [18]. Another important component is how the repeated measure analysis was implemented (e.g., platform, variance structure). However, this information is usually missing. This missing information not only triggers concerns about whether the analysis had been appropriately conducted, but also creates a gap between investigator and reader. We strongly suggest the authors should provide more information to show how the analysis was implemented (e.g., platform, procedure, variance structure) and provide the rationale that the analysis was appropriately done (e.g., provide residual plot to show that the heterogeneous variance structure assumption is met before fitting this heterogeneous variance structure in the model). The authors should also make the code used for analysis available in order to enhance the reproducibility of the results.

One critical assumption is homogeneity of variance over time. Assessment of homogeneity of variance is only relevant to RM analysis and is often set as the default algorithm for software statistical packages. The results of our simulation study demonstrate the need to assess this assumption and the need to adjust the analysis approach if not valid. Evaluation of the assumption is relatively straightforward by evaluating the observed deviation of observations at each time point. If the spread increases over time, then the homogeneity assumption is likely violated. Fortunately, many authors do plot or tabulate these data already, so the information is readily available. As shown in Figs 1–4 under the condition of heterogeneous variance, a RMA with homogeneous variance structure is not appropriate to control type I error or type II error rates. This finding was consistent regardless of covariance size or structure and group size. Our simulations were based on body weight data extracted from Cho et al. [6], which has variation that was increasing over time and these data are representative of the data observed in many preclinical animal experiments. The direction of error varies based on the time point and the true treatment effect. Based on the data extracted from Cho et al. [6], if analyzed with the inappropriate homogeneous variance structure, the estimated variance is inflated at earlier time points, leading to wide confidence intervals in treatment effect sizes and thus less powerful tests. On the contrary, the estimated variance with an incorrect homogeneous variance structure caused shrinkage of the variance and, leading to narrow confidence intervals, larger test statistics and increased type I error rates. The Cho et al. [6] data showed increasing variance over time, and this may not always the case; however, increasing variance over time is very common in practice, such as in body weight measurements. When variance is not strictly increasing over time, similar results will still hold as long as the variance is not constant over time. We have only focused on the pattern observed for one outcome, body weight in growing animals, and although this is a common outcome, the pattern in variance and errors rates we observed are not reported as generalizable to all. Instead the simulations results illustrate that investigators should examine the patterns observed in their outcomes and determine what variance structure is appropriate for that outcome and time frame.

Another critical assumption for the single time points and the RM approaches is that of normally distributed errors assumptions [19]. As discussed by Oberfeld and Frank [20], for non-normal data, repeated-measure analysis shows clear deviations from the nominal type I error rate in many conditions, even for sample sizes greater than 50.

Even though the same data with repeated measure design can be analyzed differently (RM ANOVA or Separate ANOVA), the investigator should be aware that the analysis for group differences at a specific time point or the analysis of repeated measures over time answers different research questions. For example, over a longer period one group might have a more favorable outcome than a second group. But at a specific time point after start of therapy the outcomes might be similar between groups. If the research question specifically aims to compare the treatment effect at a specific time point (e.g., the group difference at 10 weeks only is specially of interest), single-time point ANOVA at this time point is a legitimate approach to analysis. However, if the true hypothesis of interest is the treatment effect at multiple time points, then single-time point ANOVA at each time point is not recommended. Our simulation result consistently show that separate ANOVA method yields lower statistical power for detecting the treatment effect at each of the multiple time points if compared to a repeated measure analysis method for specific time point analysis. Colloquially, we can think of the repeated measures analysis as borrowing information from the other time points. Alternatively, if the hypothesis focuses on comparing groups over time across the whole process, repeated measure analysis is more desirable approach. Obviously, this does require a prespecified hypothesis and research protocol that specifies the outcome of interest. Consequently, we recommend that repeated measure analysis should be implemented regardless of the goal which is to understand treatment effect over time or treatment effect at a specific time point if qualified data is available.

For some designs and research questions it is reasonable that investigators would not include all time points in their analysis due to the concern of the data quality. For example, the researchers might not use all time points in the study for a variety of reasons, including sample size attrition due to mortality or limitations with data quality at certain time points [21]. Additionally, researcher might excluded certain time points for statistical reasons for example if the data distribution of the time point of interest was different than other time points (e.g., time points 1-5 were normal distributed but the time point 6 is of interest and it has a bimodal distribution).

Other issues related to repeated measure analysis such as multiple testing, missing value also should be aware by researcher. Even though multiple testing correction [22] is not the focus in this paper, the impact of multiplicity in the discovery framework of many preclinical animal experiments should be considered when interpreting findings [23, 24]. Missing data is another common problem in preclinical repeated measures designs and the application of multilevel models over repeated measures ANOVAs for datasets with missing values are specifically discussed in [25]. A sphericity corrections (e.g. Greenhouse-Geisser) should be specifically considered when repeated measure ANOVA is implemented either PROC GLM in SAS and ezANOVA in R [25–27].

The different approaches to analysis (no RMA or RMA) inherently lead to different reporting styles. For the RMA studies, the overall treatment, time, and treatment-by-time interaction should be expected to be reported first, and then treatment effect at individual time points should follow. For the main effects and the treatment effect at each time point, the effect size and precision should be provided in addition to any hypothesis testing. This approach to reporting provides a comprehensive summary of the findings and maximizes the information obtained from the animals in the discovery phase of research. Unfortunately, not all reviewed RMA studies have both components (overall and individual test results). Partial information may be missing, such as having no individual test results e.g., [28–30]. It is not surprising that reporting was incomplete as poor reporting is common in preclinical animal studies hence the need for reporting guidelines like ARRIVE [12, 13].

In contrast to RMA, the studies in NoRMA directly reported the significance at specific time points. This style of reporting is expected because researchers analyzed the data separately using a subset of the data at each time point. However, regrettably the reporting of partial results was common and the majority of these reported results were significant, suggesting selective reporting of significant results. This reporting strategy without clarification created two alternative questions: was the lack of reporting due to testing at selected time points or was it due to non-significance? Again, such selective reporting reduces the amount of information available for translational use of the study results and does not maximize the value of resources, including animals used in the study [31, 32].

The impact of poor reporting and analysis of other design elements such as approaches to allocation (random or not) and outcome assessment (blinded or not) on the reproducibility of research has been discussed in numerous studies [31, 33–35]; however, we are unaware of prior studies that have evaluated the reporting of repeated measures designs and the impact of including but ignoring the repeated measurement design element in preclinical research. As repeated measures create non-independent observations which has implications for error estimation, which can, in turn, impact inferential statistics based on error estimates (e.g., *p*-values) it is important to understand how and why investigators are using a repeated measures design element and any role this might play in the reproducibility of results from preclinical animal research. Meta-factor analysis was used to explore whether there is association between meta-factors and consistency of experimental design. The association was clearly identified: articles that used RMA tended to be published more recently, in journals with higher impact factor in the published year, and to have more citations in the first 24 months’ post publication. This association illustrates that consistent experimental design is also important factor associate with journal impact factor and paper citation, thus both researcher and journal reviewer should be aware to improve the impact of the paper and journal.

## Conclusion

Key elements of a repeated measures design, such as rationale, methodology, and results were frequently reported incompletely in the 58 studies with repeated-measure design, and this raises concepts of the validity of results from studies that reported repeated-measures. The findings of this study were summarized as follows:

1. Although repeated measurement of the outcome is common in preclinical experiments the rationale for inclusion is rarely reported.
2. Similarly, checking the assumption of the analysis; and covariance structure used to do the repeated-measures analysis was not described in any of the studies. Raising concerns about the reproducibility of the results.
3. Conduct of a RMA consistent with the study design and patterns of variance can control the error rates in preclinical experiments. In particular the assumption of homogeneous variance structure that is the default for many statistical packages should be assessed prior to conducting the repeated-measure analysis by checking the plots of data with a measures of variation. If this assumption is found not to be met, appropriate adjustments to the statistical models must be considered. This might be applying sphericity corrections (e.g. Greenhouse-Geisser) for repeated measures ANOVAs or incorporation of nonhomogenous covariance structures (e.g. AR1) within the model specification of multilevel models.

## Supporting information

S1 Fig Body weight from week 1 to week 28 of Figure 2 of Cho et al. [6]. Diet abbreviations: RV, the AIN-93G diet with the recommended vitamins; HFol, RV+10-fold the folate content. Gestational and pup diets denoted before and after the dash line, respectively. Weight Gain: Diet (*p*-value = 0.03), Time (*p*-value<0.0001), Diet*Time (*p*-value = 0.7). ab Significantly different by PROC MIXED model repeated measures. Values can be read are mean SEM for each treatment and time combination, n = 11-12/group.
(JPG)

S2 Fig Meta-factor analysis on BW-dataset. Meta factor for RMA-BW and NoRMA-BW in“BW-dataset”, studies with body weight as one of outcome with the repeated measurements. RMA-BW: studies reported using repeated measures analysis in the “BW-dataset”; NoRMA: studies from the “BW-dataset” did not report using repeated-measures analysis. JIP-2016: Journal impact factor in 2016; JIP-publised year: Journal impact factor in the publised year; 1st 24months’ citation: the number of the first 24 months’ citation after publication.
(TIFF)

S1 Table RMA summary. (PDF)

S2 Table NoRMA summary. (PDF)

S3 Table Meta factors data for “BW-dataset”. RMA-BW: studies reported using repeated measures analysis in the “BW-dataset”; NoRMA: studies from the “BW-dataset” did not report using repeated-measures analysis.
(PDF)

S4 Table The meta-factors association analysis for the “BW-dataset”. RMA-BW: studies reported using repeated measures analysis in the “BW-dataset”; NoRMA: studies from the “BW-dataset” did not report using repeated-measures analysis.
(PDF)
