# Supplementary figures and images for: Reporting and analysis of repeated measurements in preclinical animals experiments
Source: PLoS One. 2019 Aug 12;14(8):e0220879. doi: 10.1371/journal.pone.0220879 (PMC6690515; doi:10.1371/journal.pone.0220879)

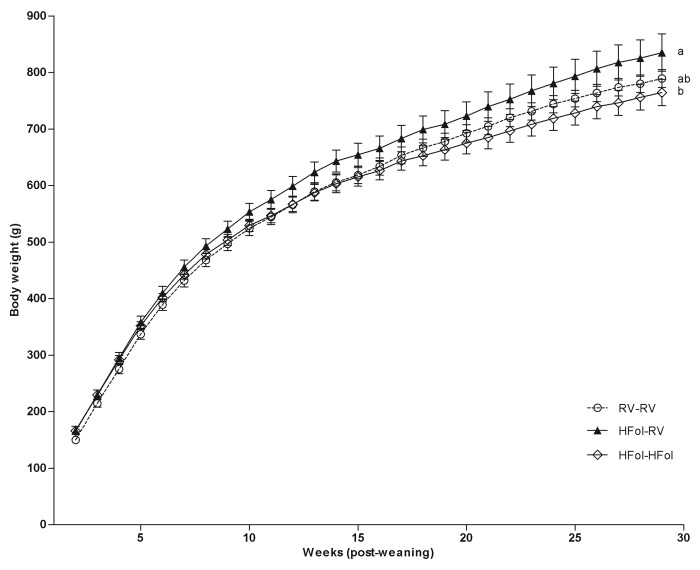

Supplement: S1 Fig — Diet abbreviations: RV, the AIN-93G diet with the recommended vitamins; HFol, RV+10-fold the folate content. Gestational and pup diets denoted before and after the dash line, respectively. Weight Gain: Diet (p-value = 0.03), Time (p-value<0.0001), Diet*Time (p-value = 0.7). ab Significantly different by PROC MIXED model repeated measures. Values can be read are mean SEM for each treatment and time combination, n = 11-12/group. (JPG) [file pone.0220879.s001.jpg]

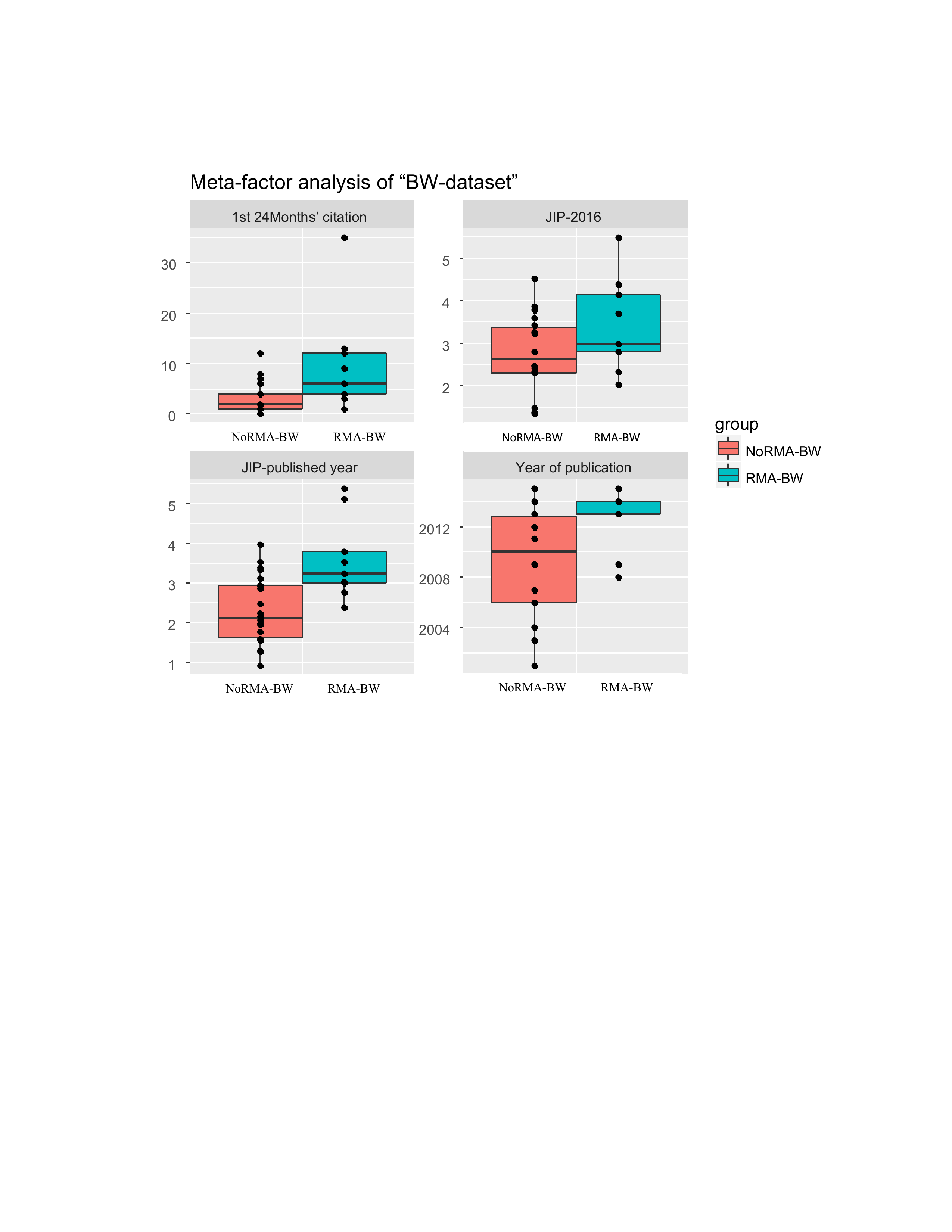

Supplement: S2 Fig — Meta factor for RMA-BW and NoRMA-BW in“BW-dataset”, studies with body weight as one of outcome with the repeated measurements. RMA-BW: studies reported using repeated measures analysis in the “BW-dataset”; NoRMA: studies from the “BW-dataset” did not report using repeated-measures analysis. JIP-2016: Journal impact factor in 2016; JIP-publised year: Journal impact factor in the publised year; 1st 24months’ citation: the number of the first 24 months’ citation after publication. (TIFF) [file pone.0220879.s002.tiff]
