# Supplementary material for: Reporting and analysis of repeated measurements in preclinical animals experiments
Source: PLoS One. 2019 Aug 12;14(8):e0220879. doi: 10.1371/journal.pone.0220879 (PMC6690515; doi:10.1371/journal.pone.0220879)
Supplement: S1 Table — (PDF) [file pone.0220879.s003.pdf]

S1 Table. RMA summary

Studies	RM Rationale	Materials & Methods						Results									
												Overall test				Individual Test	
		Normality	Equal Variance	How RMA described?	# of treatment	Repeated measure	# of time point	Outcomes	Figure	Table	Precision	F value	Treatment	Time	Treatment x time	Yes/No	# of test
Liu et al.,2014	no	no	no	Repeated measurement test	4	Body weight	7	body weight	yes	no	yes	no	yes	no	no	no	0
						Locomotion	6	locomotion									
						Rearing	6	Rearing									
						Latency	5	Latency									
Whitford et al.,2009	yes	no	no	Repeated-measures ANOVA	4	Number of lever response on FR	5	Group responsees per seession	yes	no	yes	no	yes	yes	yes	no	0
						Efficiency ration for DRL	10	Group efficiency ratios									
Bera et al.,2007	no	no	no	Two way ANOVA for repeated measures	3	Pup weight	NA	Pup weight gain	no	yes	yes	no	yes	no	no	no	NA
						Locomotor activity test	4	Locomotor activity test		no	NA			no	no	NA	
						Conditioned avoidance response (CARs)	3	CARs	yes		yes			yes	yes	yes	3
						Blood pressure		Blood pressure						no	no	NA	NA
Jin et al.,2008	no	yes	yes	(TW-RM-ANOVA)	15	Urine volume	3	Urine volume	no	NA	no	no	yes	no	no	yes	NA
						Osmolality		osmolality;		yes	yes						3
						Creatinine		Creatinine									
						GGT		GGT									
Basha and Sujitha,2012	no	no	no	TW-RM-ANOVA	6	Mean of correct choices/session	9	Mean of correct choices(session)	yes	no	no	no	yes	yes	yes	no	0
						Latency change	8	Latency change									
Basha et al.,2011	no	no	no	Two-factor ANOVA with RM	7	Number of alternation	NA	No. of correct choice/10 trials	no	no	no	no	yes	yes	yes	no	0
						Latency		Latency									
Black et al., 2011	no	yes	yes	Repeated measures analysis of variance	4	Grip stength	2	Grip stength	yes	no	yes	no	yes	no	no	no	0
Sitting et al.,2012	no	no	no	Two-way repeated measures ANOVA	2	Latency to reach platform	4	Latency to reach platform	yes	no	yes	no	yes	yes	yes	yes	4
						Mean swim speed		Mean swim speed									
						Distance traveled		Distance traveled									
Liu et al., 2010	no	no	no	Test by repeated measure	3	Escape latency	6	Escape latency	no	yes	yes	no	yes	no	no	yes	6
Carvalho et al.,2007	no	no	no	Repeated measures and ANOVA	4	Liquid consumption	17	Liquid consumption	no	no	yes	no	no	no	no	no	0
						Solid comsumption		Solid comsumption									
Jensen et al.,2013	no	no	no	Repeated measures ANOVA	6	Mean latency (MWM)	NA	Mean latency (MWM)	yes	no	yes	yes	yes	yes	yes	no	0
						Mean latency (RMWM)	NA	Mean latency (RMWM)									
Carlin et al.,2013	no	no	no	Repeated measures by time	4	Maternal weight gain	NA	Maternal weight gain	no	yes	yes	no	yes	no	yes	yes	3
						Offspring weight gain		Offspring weight gain	yes	no							4
Ma et al.,2015	no	no	no	Repeat measurement test	3	Body weight	7	Body weight	no	yes	yes	no	no	no	no	no	0
						Von Frey hairs test	5	Withdrawal threshold	yes	no						yes	5
						FT	6	Finches/10 mins									6
								licking time/ 10 mins									
Burdge et al.,2008	no	yes	no	General linear Model with repeated measures	4	Dam weight	8	Dam weight	yes	no	yes	no	yes	yes	yes	yes	8
						Litter weight	5	Litter weight									5
						Offspring weight	12	body weight									12
Cho et al.,2013	no	no	no	PROC MIXED .. time as the main factors	3	Food intake	13	Food intake	yes	no	yes	no	yes	yes	yes	no	0
						Body weight gain	28	Body weight gain									
						Blood glucose response	6	Blood glucose response								yes	6
Cho et al.,2013	no	no	no	PROC MIXED time as main factor	4	Food intake	13	Food intake	yes	no	yes	no	yes	yes	yes	no	0
						Body weight gain	28	Body weight gain									
Pannia et al.,2015	no	no	no	Two way repeate measures analysis of vairance	4	Food intake	14	Cumulative FI	yes	no	yes	no	yes	yes	yes	no	0
						Pup body weight)		Changes in body weight									
Jiang et al.,2014	no	no	no	Repeated mesures design analysis of variance	4	Daily water intake	120	Daily Fluid comsumption	yes	no	yes	no	yes	yes	yes	yes	12
						Body weight	12	Body weight									
						Escape Latency	5	Escape Latency	no	yes							5
Bartos et al.,2015	no	no	no	Repeated-measures ANOVA	3	Number of squares	3	Number of squares	yes	no	yes	yes	yes	yes	yes	no	0
						Number of rearings		Number of rearings									

Burdge et al., 2009	yes	no	no	General linear Model with repeated measures	4	Change in Body weight	9	Change in Body weight	yes	no	yes	no	no	no	yes	yes	9
Day et al.,2005	yes	no	no	Repeated measures ANOVA	6	Grip strength in F0	4	Grip strength in F0	yes	no	yes	no	yes	yes	yes	yes	4
						Grip strength in F1	2	Grip strength in F1								no	0
						Runing wheel in F1	3	Runing wheel in F1									
Paletz et al.,2006	no	no	no	Univariate (RMA-NOVA)	6	Response rate	NA	response rates	yes	no	yes	no	yes	yes	yes	yes	15
						Response efficiency	NA	Response efficiency									2
Reed et al.,2006	no	no	no	Repeated-measures analysis of variance (RMANOVA)	6	Correct	NA	Correct	yes	no	yes	no	yes	yes	yes	yes	NA
						Erros		Erros									
						Omission trails		Omission trails									
						Rearing lever latency		Rearing lever latency									
						Choice latency		Choice latency									
