# Supplementary material for: Reporting and analysis of repeated measurements in preclinical animals experiments
Source: PLoS One. 2019 Aug 12;14(8):e0220879. doi: 10.1371/journal.pone.0220879 (PMC6690515; doi:10.1371/journal.pone.0220879)
Supplement: S2 Table — (PDF) [file pone.0220879.s004.pdf]

## S2 Table. NoRMA summary

| | | Materials & Methods | | | | | | Results | | | | Individual Test | |
|---|---|---|---|---|---|---|---|---|---|---|---|---|---|
| Studies | RM rationale | Normality | Equal variance | How Analysis Described | # of treatment | Repeated Measures | # of time point | Outcome(s) | Figure | Table | Precision | Yes/No | # of test |
| Cheng et al., 2009 | no | no | no | analysis of variance (ANOVA) | 3 | Escape latencies | 3 | Escape latencies | yes | no | yes | yes | 3 |
| Balayssac et al., 2002 | no | no | no | analysis of variance | 4 | Withdrawal letency | NA | Withdrawal letency | yes | no | yes | yes | 24_SD rats; 29_Lou/C rats |
| | | | | | | Withdraw threshold | | Withdraw threshold | | | | | 16_SD rats;20_Lou/C rats |
| Niu et al., 2009 | no | no | no | one-way ANOVA | 4 | Learning day | 4 | Learning day | no | yes | yes | yes | 4 |
| | | | | | | Error number | | Error number | | | | | |
| | | | | | | Total reaction time | | Total reaction time | | | | | |
| Wu et al., 2006 | no | no | no | not described | 4 | Error number (1) | 3 | Error number (1) | no | yes | yes | yes | 3 |
| | | | | | | Error number (2) | | Error number (2) | | | | | |
| | | | | | | Sustaining time | | Sustaining time | | | | | |
| Uchikawa et al., 2011 | no | no | no | Student's t test | 3 | Blood Hg levels | 4 | Blood Hg levels | yes | no | yes | yes | 4 |
| Shooshtari et al., 2012 | no | no | no | one-way ANOVA | 5 | Step-through latency | 2 | Step-through latency | yes | no | yes | yes | 2 |
| Folven et al., 2009 | no | yes | yes | Two-way ANOVA | 6 | Hang/climb score | 6 | Hang/climb score | yes | no | yes | yes | 4 |
| Cui et al., 2009 | no | no | no | One way analysis of vairance | 5 | Average weight | 5 | Average weight | yes | no | yes | yes | 5 |
| Salama et al., 2012 | no | no | no | one way analysis of variance (ANOVA) | 5 | Body weight | NA | Body weiht gain | no | yes | yes | no | 0 |
| | | | | | | Fluid intake | | Fluid intake | | | | | |
| | | | | | | Food intake | | Food intake | | | | | |
| Dong et al., 2009 | yes | yes | yes | one way analysis of variance (ANOVA) | 6 | Body weight | 60 | Body weight gain | yes | yes | yes | no | 0 |
| | | | | | | | | Body weight at end point | | no | | yes | 1 |
| | | | | | | Food intake | | Food intake | | yes | | no | 0 |
| Dong et al., 2011 | yes | yes | yes | one way analysis of variance (ANOVA) | 6 | Body weight | 60 | Body weight gain | no | yes | yes | no | 0 |
| | | | | | | Food intake | | Food intake | | | | yes | 1 |
| Dong et al., 2012 | yes | yes | yes | one way analysis of variance (ANOVA) | 4 | Body weight | 60 | body weight gain | no | yes | yes | no | 0 |
| | | | | | | Food intake | | Food intake in 60th day | | | | yes | 1 |
| Huang et al., 2014 | no | no | no | Analysis of viriance | 3 | body weight | 16 | body weight | yes | no | yes | yes | 16 |
| | | | | | | Food intake | 13 | Food intake | | | | | 13 |
| | | | | | | Blood glucose | 5 | Blood glucose | | | | | 5 |
| Farina et al., 2003 | no | no | no | one-way ANOVA | 6 | Liquid ingestaion | 15 | Liquid ingestaion | no | yes | yes | no | 0 |
| | | | | | | Body weight | | Body weight gain | | | | | |
| Sable et al.,2011 | no | no | no | one way ANOVA | 6 | Dam body weight | 4 | Dam Body weight gain | no | no | no | no | 0 |
| | | | | | | Food intake | 20 | | | | | | |
| Sable et al., 2012 | no | no | no | one way ANOVA | 11 | Body weight | 4 | Average pup weight | no | yes | yes | yes | 4 |
| | | | | | | Food intake | 21 | Average daily Food intake | | no | | no | 0 |
| Joshi et al., 2004 | no | no | no | One way analysis of vairance | 4 | Dam weight | 4 | Dam Weight gain | no | yes | yes | no | 0 |
| | | | | | | Pup weight | NA | Pup weight | | | | yes | 9 |
| Field et al., 2006 | no | no | no | one or two way ANOVA | 3 | Body weight | 10 | Body weight | no | yes | yes | yes | 1 |
| | | | | | | | | Weight change | | | | no | 0 |
| | | | | | | Food intake | | food intake | | | | | |

| | | | | | | | | | | | | | |
|---|---|---|---|---|---|---|---|---|---|---|---|---|---|
| Sarkozi et al., 2015 | yes | yes | no | Parametic one-way ANOVA | 4 | body weight | 72 | body weight | yes | no | no | yes | 12 |
| | | | | | | Food and water consumption | | Food and water consumption | no | | NA | | NA |
| | | | | | | Ambulation distance | 3 | Ambulation distance | no | yes | yes | yes | 3 |
| | | | | | | Ambulation time | | Ambulation time | | | | | |
| | | | | | | Ambulation count | | Ambulation count | | | | | |
| | | | | | | Local activity time | | Local activity time | | | | | |
| | | | | | | Local activity count | | Local activity count | | | | | |
| | | | | | | Immobility time | | Immobility time | | | | | |
| | | | | | | Immobility count | | Immobility count | | | | | |
| | | | | | | Rearing time | | Rearing time | | | | | |
| | | | | | | Rearing count | | Rearing count | | | | | |
| | | | | | | Percent of time in Corner zones | | Percent of time in Corner zones | | | | | |
| | | | | | | Percent of time in Side zones | | Percent of time in Side zones | | | | | |
| | | | | | | Percent of time in Central zones | | Percent of time in Central zones | | | | | |
| Bey-routy and Chan,2006 | no | no | yes | Analysis of vairance | 5 | Dam weight in F0 | NA | Body weight gain in F0 | no | yes | yes | no | 0 |
| | | | | | | Food consumption in F0 | | Food consumption in F0 | | | | | |
| | | | | | | Pup weight in F1 | | Pup weight in F1 | | | | yes | 7 |
| Chmurzyn-ska et al., 2012 | no | no | yes | fixed model ANOVA | 4 | Dam food intake | NA | Dam diet consumption | no | no | yes | no | 0 |
| | | | | | 8 | Dam weight gain | | overall weight gain | | | | | |
| | | | | | | Pup food intake | 140 | Food intake | | | | yes | 2 |
| | | | | | | Pup weight gain | 20 | Pup weight | yes | | | | |
| Nassiri-Asl et al.,2011 | no | no | yes | one-way ANOVA | 5 | Step through latency | 3 | Step through latency | yes | no | yes | yes | 3 |
| Banji et al.,2013 | no | no | no | ANOVA | 5 | Serum fluride levels | 2 | Fluoride levels in serum | no | yes | yes | yes | 2 |
| | | | | | | mid-air righting attempts | 5 | NO. capable of righting in mid air | | | | | 5 |
| Balaji et al., 2015 | no | no | no | one-way analysis of variance (ANOVA) | 7 | Body weight | 4 | Weekly Body weight | no | no | NA | NA | NA |
| | | | | | | daily warter/feed intake | 30 | Warter/feed consumption | | | | | |
| Giudi-celli et al.,2013 | no | no | yes | Kruskall-Wallis test | 4 | Dam weight | NA | Dam weight | no | yes | yes | yes | 3 |
| | | | | | | Pup weight | 93 | Pup weight | yes | no | | | 25 |
| Jin et al., 2007 | yes | yes | yes | Two-way ANOVA | 15 | Food intake | 11 | Food intake | yes | yes | yes | no | 0 |
| | | | | | | body weight | 18 | body weight | | | | yes | 1 |
| El-lethey et al.,2010 | no | no | no | anlyses of variance (ANOVA) | 5 | Body weight | 11 | Average body weight gain | yes | no | no | no | 0 |
| | | | | | | number of squares crossed | 3 | number of squares crossed | | | | | |
| | | | | | | number of vertical activity | 3 | number of vertical activity | | | | | |
| | | | | | | Freezing time | 3 | Freezing time | | | | | |
| | | | | | | Latency | 5 | Latency | | | | | |
| | | | | | | Number of entries of blind alleys | 5 | Number of entries of blind alleys | | | | | |
| | | | | | | numbers of rears | 2 | numbers of rears | | | | | |
| | | | | | | numbers of head dips | 2 | numbers of head dips | | | | | |
| Ekam-baram and Paul, 2001 | no | no | no | one-way analysis of variance (ANOVA) | 4 | Food intake | 60 | Average Food intake | yes | no | yes | no | 0 |
| | | | | | | Body weight | 2 | Body weight gain | | | | | |
| Ekam-baram et al.,2003 | no | no | no | one way analysis of variance (ANOVA) | 4 | Food intake | 60 | Average Food intake | yes | no | yes | no | 0 |
| | | | | | | Body weight | 2 | Body weight gain | | | | | |

| | | | | | | | | | | | | | |
|---|---|---|---|---|---|---|---|---|---|---|---|---|---|
| Achon et al., 1999 | no | no | no | Two-way ANOVA | 4 | Dietary intake | NA | Food intake | yes | no | yes | yes | 9 |
| | | | | | | Body weight | | Body weight | | | | | 10 |
| | | | | | | Fat | | MPU/NPU | no | yes | | no | 0 |
| | | | | | | | | Nitrogen intake | | | | | |
| | | | | | | Nitrogen | | Fecal nitrogen | | | | | |
| | | | | | | | | Urina nitrogen | | | | | |
| | | | | | | | | NDC | | | | | |
| Butenhoff et al.,2004 | no | no | yes | Analysis of variance (ANOVA) | 5 | Body weight at P1 | 12 | NA | no | no | NA | NA | NA |
| | | | | | | Feed consumption at P1 | | | | | | | |
| | | | | | | Body weight at F1 | NA | Mean pup weight/litter | no | yes | yes | yes | 5 |
| | | | | | | Body weight at F2 | 5 | | | | | | |
| Marvin et al.,1950 | no | no | no | Mean+SE | 9 | Body weight | 4 | Body weight | yes | yes | yes | no | 0 |
| Lucena et al., 2007 | no | no | no | one-way ANOVA | 5 | Liquid and solid ingestions | 14 | Liquid and solid ingestions | NA | NA | NA | NA | NA |
| Cordero et al.,2013 | no | no | no | Two-way ANOVA | 4 | Fat mass | 3 | Body fat mass | no | yes | yes | yes | 1 |
| EI-lethey and Kamel, 2011 | no | no | no | Means of analyses of vairance (ANOVA) | 4 | Body weight | 15 | Body weight gain | no | yes | yes | no | 0 |
