# Supplementary material for: Reporting and analysis of repeated measurements in preclinical animals experiments
Source: PLoS One. 2019 Aug 12;14(8):e0220879. doi: 10.1371/journal.pone.0220879 (PMC6690515; doi:10.1371/journal.pone.0220879)
Supplement: S3 Table — RMA-BW: studies reported using repeated measures analysis in the “BW-dataset”; NoRMA: studies from the “BW-dataset” did not report using repeated-measures analysis. (PDF) [file pone.0220879.s005.pdf]

S3 Table. Meta factors data in "BW-dataset".

Studies of repeated measure in BW	Repeated mesure anslysis described?	Category	Year of publication	First 24 months' citations	Journal impact factor in 2016	Journal impact factor in the publised year	Guidline of journal reported?
Burdge et al., 2008	yes	RMA-BW	2008	4	3.71	2.76	yes
Burdge et al., 2009	yes	RMA-BW	2009	35	4.14	3.79	yes
Carlin et al., 2013	yes	RMA-BW	2013	12	2.81	3.53	yes
Cho et al., 2013	yes	RMA-BW	2013	13	4.39	5.11	yes
Cho et al., 2013	yes	RMA-BW	2013	6	5.49	5.39	yes
Liu et al., 2014	yes	RMA-BW	2014	4	2.34	3.03	yes
Jiang et al., 2014	yes	RMA-BW	2014	9	2.81	3.23	yes
Ma et al., 2015	yes	RMA-BW	2015	3	2.05	2.38	no
Pannia et al., 2015	yes	RMA-BW	2015	1	3.00	3.00	yes
Ekambarma and Paul, 2001	no	NoRMA-BW	2001	0	1.34	0.91	yes
Ekambaram et al., 2003	no	NoRMA-BW	2003	0	2.31	1.27	yes
Farina et al., 2003	no	NoRMA-BW	2003	4	3.86	2.22	yes
Joshi et al., 2004	no	NoRMA-BW	2004	2	3.42	1.96	yes
Butenhoff et al., 2004	no	NoRMA-BW	2004	0	3.58	2.06	yes
Beyrouty and Chan, 2006	no	NoRMA-BW	2006	6	2.41	2.14	yes
Field et al., 2006	no	NoRMA-BW	2006	0	4.52	2.94	no
Jin et al., 2007	no	NoRMA-BW	2007	7	3.58	2.92	yes
Niu et al., 2009	no	NoRMA-BW	2009	2	2.31	1.29	yes
Cui et al., 2009	no	NoRMA-BW	2009	1	2.47	1.74	no
Dong et al., 2009	no	NoRMA-BW	2009	6	2.47	1.74	no
Dong et al., 2011	no	NoRMA-BW	2011	2	2.47	1.93	yes
Sable et al., 2011	no	NoRMA-BW	2011	4	3.28	3.38	no
Sable et al., 2012	no	NoRMA-BW	2012	8	3.28	3.12	no
Chmurzynska et al., 2012	no	NoRMA-BW	2012	1	2.80	3.33	yes
Dong et al., 2012	no	NoRMA-BW	2012	2	3.79	3.98	yes
Salama et al., 2012	no	NoRMA-BW	2013	0	1.38	1.55	yes
Giudicelli et al., 2013	no	NoRMA-BW	2013	2	2.81	3.53	yes
Huang et al., 2014	no	NoRMA-BW	2014	12	3.23	2.86	yes
Balaji et al., 2015	no	NoRMA-BW	2015	1	1.38	1.55	yes
Sarkozi et al., 2015	no	NoRMA-BW	2015	1	1.49	1.58	no
Bartos et al., 2015	no	NoRMA-BW	2015	2	2.34	2.46	yes
