# Supplementary material for: Reporting and analysis of repeated measurements in preclinical animals experiments
Source: PLoS One. 2019 Aug 12;14(8):e0220879. doi: 10.1371/journal.pone.0220879 (PMC6690515; doi:10.1371/journal.pone.0220879)
Supplement: S4 Table — RMA-BW: studies reported using repeated measures analysis in the “BW-dataset”; NoRMA: studies from the “BW-dataset” did not report using repeated-measures analysis. (PDF) [file pone.0220879.s006.pdf]

**S4 Table. The meta-factors association analysis for the "BW-dataset".**

| Categories | RMA-BW | | | NoRMA-BW | | | RMA-BW vs NoRMA-BW |
|---|---|---|---|---|---|---|---|
| | Range | Mean | SD | Range | Mean | SD | Wilcoxon Test $p-$value |
| | | | | | | | P value |
| Year of publication | 2008-2015 | 2013 | 2.5 | 2001-2015 | 2009 | 4.4 | 0.0396 |
| The first 24 months' citations | 1-35 | 9.67 | 10.34 | 0-12 | 2.86 | 3.15 | 0.096 |
| Journal impact factor in 2016 | 2.05-5.49 | 3.41 | 1.11 | 1.34-4.52 | 2.75 | 0.88 | 0.1632 |
| Journal impact factor in the published year | 0.91-3.98 | 3.58 | 1.03 | 2.38-5.39 | 2.29 | 0.84 | 0.0047 |
